# Inhibitory effect of *Bifidobacterium infantis*-mediated sKDR prokaryotic expression system on angiogenesis and growth of Lewis lung cancer in mice

**DOI:** 10.1186/1471-2407-12-155

**Published:** 2012-04-26

**Authors:** Zhao-Jun Li, Hong Zhu, Bu-Yun Ma, Fen Zhao, Shu-Hua Mao, Tai-Guo Liu, Jian-Ping He, Li-Cong Deng, Cheng Yi, Ying Huang

**Affiliations:** 1 Department of Abdominal Cancer, West China Hospital, Sichuan University, Chengdu, 610041, Sichuan Province, China; 2 Division of Ultrasonography, West China Hospital, Sichuan University, Chengdu, 610041, Sichuan Province, China; 3 Department of Pathophysiology, West China School of Preclinical and Forensic Medicine, Sichuan University, Chengdu, 610041, Sichuan Province, China; 4 Department of Oncology, Center Hospital, Bazhong, 636000, Sichuan Province, China

## Abstract

**Background:**

To construct the *Bifidobacterium infantis*-mediated soluble kinase insert domain receptor (sKDR) prokaryotic expression system and to observe its inhibitory effect on growth of human umbilicus vessel endothelial cells (HUVECs) *in vitro* and Lewis lung cancer (LLC) on mice *in vivo.*

**Methods:**

The *Bifidobacterium infantis*-mediated sKDR prokaryotic expression system was constructed through electroporation and subsequently identified through PCR and Western blot analysis. HUVECs were added to the products of this system to evaluate the anti-angiogenesis effect through MTT assay *in vitro*. The LLC mice models were divided into three groups: one group treated with saline (group a); one group treated with recombinant *Bifidobacterium infantis* containing pTRKH2-PsT plasmid group (group b); and one group treated with recombinant *Bifidobacterium infantis* containing pTRKH2-PsT/sKDR plasmid group (group c). The quality of life and survival of mice were recorded. Tumor volume, tumor weight, inhibitive rate, and necrosis rate of tumor were also evaluated. Necrosis of tumor and signals of blood flow in tumors were detected through color Doppler ultrasound. In addition, microvessel density (MVD) of the tumor tissues was assessed through CD31 immunohistochemical analysis.

**Results:**

The positively transformed *Bifidobacterium infantis* with recombinant pTRKH2-PsT/sKDR plasmid was established, and was able to express sKDR at gene and protein levels. The proliferation of HUVECs cultivated with the extract of positively transformed bacteria was inhibited significantly compared with other groups (P < 0. 05). The quality of life of mice in group c was better than in group a and b. The recombinant *Bifidobacterium infantis* containing pTRKH2-PsT/sKDR plasmid enhanced the efficacy of tumor growth suppression and prolongation of survival, increased the necrosis rate of tumor significantly, and could obviously decrease MVD and the signals of blood flow in tumors.

**Conclusion:**

The *Bifidobacterium infantis*-mediated sKDR prokaryotic expression system was constructed successfully. This system could express sKDR at gene and protein levels and significantly inhibit the growth of HUVECs induced by VEGF *in vitro*. Moreover, it could inhibit tumor growth and safely prolong the survival time of LLC C57BL/6 mice.

## Background

The growth, invasion, and metastasis of malignant tumors are angiogenesis-dependent processes [1,2]. The anti-angiogenesis study of tumor has become increasingly popular after Folkman first proposed in 1971 that tumor growth depends on new blood vessels [3,4]. To date, anti-angiogenesis therapy has been proven effective by many studies and has become an irreplaceable method for cancer treatment [4-8].

Vascular endothelial growth factor (VEGF) is the most important factor for angiogenesis; it is secreted abundantly in tumor tissues [9-11]. VEGF can bind specifically to VEGFR (mainly VEGFR1 and VEGFR2) and induce blood vessel formation. The blockage of this binding process may significantly suppress the formation of new blood vessels in the tumor, and then suppress tumor growth, invasion, and metastasis [12,13]. The soluble kinase insert domain receptor (sKDR) is a soluble form of the extramembrane part of VEGFR2 [14]. It has the same high affinity for VEGF but does not induce angiogenesis [15]. Thus, the delivery of sKDR to tumor tissues inhibits the formation of new blood vessels in the tumor tissues and has an anti-tumor effect [6,16].

Deficiency of the specifically targeted vector is an important obstacle in cancer gene therapy [17]. Although viruses and liposomes have been widely used as delivery vectors, their lack of specific targeting property causes massive distribution to untargeted tissues and results in several side effects [18-21]. Thus, developing specific targeting vectors is very important.

*Bifidobacterium infantis* is an anaerobic and non-pathogenic bacterium that has been proven by our previous study to specifically target the anaerobic environment of tumor center [22]. The aim of this study was to construct a *Bifidobacterium infantis-*mediated sKDR prokaryotic expression system and to observe its effect on human umbilicus vessel endothelial cells (HUVECs) *in vitro* and on Lewis lung cancer (LLC) mice model *in vivo*.

## Methods

### Materials

Strains of *Bifidobacterium infantis* 2001 were obtained from the Key Laboratory of West China School of Stomatology, Sichuan University (China). PTRKH2-PsT plasmid containing erythromycin^r^ (Ery^r^) gene was provided by the Life Scientific College of Fudan University (China). The LLC cell line was provided by the State Key Laboratory of Biomedicine, Sichuan University (China). Recombinant *E. coli* DH5α line containing pcDNA3.1/sKDR was constructed in our laboratory [23]. Female C57BL/6 mice (6 weeks to 8 weeks old) weighing between 16 and 18 g were purchased from the Experimental Animal Center of Sichuan University (China). DNA Marker III was purchased from Tiangen (China) or Transgen (China). T4 DNA ligase, *Bam*H I, *Sal* I, and One-Step RNA PCR Kit were purchased from Takara (Dalian, China). The plasmid purification kit, PCR product purification kit, plasmid mini preparation kit, Wizard PCR Preps DNA Purification System, and gel extraction kit were purchased from Omega (USA). Mouse monoclonal antibody for sKDR was purchased from R&D (USA). Polyvinylidene difluoride (PVDF) membranes were purchased from Millipore (USA). The PCR reaction test kit was purchased from Tiangen (China). Trizol reagent was purchased from Transgen (China). 3-(4,5-Dimethylthiazol-2-yl)-2, 5-diphenyltetrazolium bromide (MTT), lysozyme, and trypsin were purchased from Sigma (USA). Mouse monoclonal antibody for CD31 and LSAB kit were purchased from Dako (Denmark). The enhanced chemiluminescence (ECL) detection kit and X-ray films were purchased from Roche (Switzerland). Dulbecco's modified Eagle's medium (DMEM), M199 medium, and dimethylsulfoxide (DMSO) were purchased from Gibco (USA).

### Construction of recombinant plasmid

Strains of *E. coli* DH5α containing plasmid pTRKH2-PsT were inoculated into 5 ml LB liquid medium with 300 μg/ml Ery and shaken overnight at 37°C for 12 hours. Subsequently, plasmid pTRKH2-PsT was purified using a plasmid purification kit (Omega Co.). Specific primers of sKDR gene were designed based on published sequences (GenBank, AF063658). The upstream primer was 5’-CCGGGATCCATGGAGAGCAAGGTGCTG-3’ and the downstream primer was 5’-GTGGTCGACTTTTTCATGGACCCTGAC-3’. After an initial denaturation for 2 min at 94°C, 3.0 μl DNA template was amplified for 35 cycles of denaturation at 94°C for 30 s, annealed at 55°C for 30 s, with an extension at 72°C for 2 min, followed by a final extension for 8 min at 72°C. The amplified products were confirmed via electrophoresis on 0.9% agarose gel. Approximately 9 μl sKDR gene and 3 μl pTRKH2-PsT plasmid were digested by two restriction enzymes (*Bam*H I and *Sal* I*),* joined by T4 DNA ligase at 16°C for 12 h, and finally stored at −20°C. The recombinant pTRKH2-PsT/sKDR plasmid was then separated through electrophoresis to confirm whether the ligation products had the desired size.

### Transformation of bifidobacterium infantis by electroporation

*Bifidobacterium infantis* were harvested and washed after growth at 37°C and resuspended in 40 μl ice-chilled sucrose solution with 1 mmol/L ammonium citrate. About 40 μl suspension and electroporation was carried out to transfect recombinant pTRKH2-PsT/sKDR into *Bifidobacterium infantis* at 25 μF, 2.0 kV. The cuvette was connected parallel to a 200 Ω resistor.

### Screening and identification of positive clones

The transformed *Bifidobacterium infantis* were cultivated in MRS plates with 10 μl Ery until the colonies achieved 1 mm diameter. A single positively transformed colony was selected and incubated at 37°C in 5 ml Ery^r^ MRS liquid medium for 12 h. The plasmid DNA was extracted and digested by *Bam*H I (1 μl) and *Sal* I (1 μl) at 37°C for 4 h. The products were then identified via electrophoresis through 0.9% agarose gel. The identified recombinant plasmid DNA was used as template and amplified through PCR with specific primers of sKDR gene. The amplified products were confirmed via electrophoresis on 0.9% agarose gel. Sequencing was carried out by Invitrogen Co. (Shanghai, China).

### Detection of sKDR gene expression of recombinant positive bifidobacterium infantis

The total RNA of recombinant positive colonies was extracted using Trizol reagent. RT-PCR products were synthesized using total RNA as template (30 times amplification cycle with the condition of denaturation at 94°C for 30 s, annealing at 55°C for 30 s, and an extension at 72°C for 1 min). The amplified products were then identified through 0.9% agarose gel electrophoresis.

The positively transformed *Bifidobacteria infantis* (100 μl) were inoculated into 20 ml Ery^r^ MRS liquid medium into an anaerobic environment at 37°C for 24 h. The bacteria were then harvested via centrifugation, resuspended in lysis buffer (50 mM Tris·HCl, 2 mM EDTA, 100 mM NaCl, 0.5%Triton X-100, 1 mg/ml lysozyme, pH 8.5), and sonicated. Protein concentration was determined using the bicinchoninic acid (BCA) method. Approximately 30 μg of protein was subjected to 4% to 12% gradient SDS-PAGE using a Tris-glycine system, and then the gel was electroblotted onto PVDF membrane for 45 min. The membrane was subsequently incubated with 5% non-fat dry milk in PBS for 1 h to block nonspecific binding sites, and then incubated with the appropriate primary antibody concentration (1:200 dilution for sKDR) for 2 h at 37°C in 5% non-fat dry milk. The membrane was subsequently rinsed in PBS and then incubated for 2 h at 37°C with goat antimouse IgG-HRP at 1:2000 dilution. The membrane was rinsed and visualized with ECL detection reagents after incubation.

### Effect of recombinant bifidobacterium infantis on HUVECs

Recombinant *Bifidobacterium infantis* containing pTRKH2-PsT/sKDR and pTRKH2-PsT plasmids were broken through ultrasonic wave and purified via dialysis. HUVECs were digested with 0.25% trypsin and then cultured *in vitro.* They were then diluted and inoculated into a 96-well plate at a concentration of 1 ×10^4^ cells and a volume of 0.2 ml per well in 40 wells. The 40 wells were randomized into 4 groups after cultivated in M199 medium containing fatal bovine serum (10 wells per group): group 1 (negative control group) added with 0.2 ml liquid medium; group 2 (VEGF group) added with 0.2 ml liquid medium containing VEGF (6 ng/ml); group 3 (pTRKH2-PsT + VEGF group) added with final solution of recombinant *Bifidobacterium infantis* containing pTRKH2-PsT plasmid 0.2 ml and VEGF (6 ng/ml); and group 4 (pTRKH2-PsT/sKDR + VEGF group) added with final solution of recombinant *Bifidobacterium infantis* containing pTRKH2-PsT/sKDR plasmid (0.2 ml) and VEGF (6 ng/ml). Cells were incubated at 37°C for 24 h, and then 0.02 ml MTT (5 mg/ml final concentration) was added to each well after an additional 4 h of incubation to allow MTT to form formazan crystals by reacting with metabolically active cells. Approximately 150 μl DMSO was added to each well to dissolve the formazan crystals at 37°C for 10 min. The absorbance values of the solution in each well were measured at 490 nm using a microplate reader. Cell viability (%) = absorbance of the treated group/absorbance of VEGF group × 100%.

### Cell culture

LLC cells were cultured in Dulbecco’s modified Eagle’s medium (DMEM) supplemented with 10% fetal bovine serum plus ampicillin and streptomycin routinely, and incubated in 5% CO_2_ at 37°C.

### Animal experiment designation

All animal procedures were approved by the Animal Care and Scientific Committee of Sichuan University. The tumor tissues from LLC mice were triturated and prepared into cell suspensions (dilution 1:5 with normal saline). The cells harvested from xenografts were adjusted to a concentration of 1 × 10^7^/ml, and 0.2 ml cell suspensions were inoculated subcutaneously into the armpit of right anterior superior limbs of C57BL/6 mice. The mice were randomized into 3 groups (8 mice per group) after 7 days when the tumors were palpable (2 mm to 4 mm diameter): the control group (group a) injected with 0.4 ml saline through the caudal vein, the recombinant *Bifidobacterium infantis* containing pTRKH2-PsT plasmid group (group b) injected with 0.4 ml suspension of *Bifidobacterium infantis* containing pTRKH2-PsT plasmid (1 × 10^8^/ml) through caudal vein, and the recombinant *Bifidobacterium infantis* containing pTRKH2-PsT/sKDR plasmid group (group c) injected with 0.4 ml suspension of *Bifidobacterium infantis* containing pTRKH2-PsT/sKDR plasmid (1 × 10^8^/ml) through caudal vein. The mice were successively treated with the same dose of *Bifidobacterium infantis* every 3 days, which lasted for 21 days. The mice of the 3 groups were killed after 21 days of treatment. The tumors were then excised and weighed.

### Identification of targeting property and sKDR expression *in vivo*

Another 18 LLC bearing mice were divided into 3 groups and received the same treatment as mentioned above, and subsequently killed after 21 days of treatment. The tumor and other tissues were operated under sterile conditions. Part of these tissues was homogenized using saline into a concentration of 10%, respectively. Approximately 100 μl homogenate of all the tissues was cultured on MRS plate medium with Ery 10 μg/ml, 0.05% cysteine hydrochloride, and 0.5 M sucrose, and then incubated into an anaerobic environment at 37°C for 3 days. About 20 μg protein extracted from the tumor tissue of each group was subjected to 4% to 12% gradient SDS–PAGE using a Tris–glycine system. The gel was subsequently electroblotted onto PVDF membrane for 45 min. The membrane was incubated with 5% non-fat dry milk in PBS for 1 h to block nonspecific binding sites, and then incubated with the appropriate primary antibody concentration (1:200 dilution for sKDR) for 2 h at 37°C in 5% non-fat dry milk. The membrane was rinsed in PBS and incubated for 2 h at 37°C with goat antimouse IgG-HRP at 1:2000 dilution. The membrane was rinsed and visualized with ECL detection reagents after incubation.

### Tumor growth and inhibitive rate of tumor

The length, width, and weight of tumor were measured using a slide caliper every three days. Tumor volume (TV) was estimated using the formula: TV (mm^3^) = (width^2^ × length)/2. Inhibitive rate of tumor was calculated using the formula: inhibitive rate of tumor (%) = (1 - average weight in treated group/average weight in control) × 100% [24].

### Necrosis rate and blood flow of tumor

The remaining mice in all groups underwent color Doppler ultrasound before they were killed after 21 days of treatment. The size, tumor shape, ultrasonic echo from tumor inner to known liquefied tumor, and necrotic tissues were detected under two-dimensional ultrasound by color Doppler (ACUSON 1228ST) with 5 MHz frequency.

The necrosis rate of tumor was determined using the following formula [25]: necrosis rate of tumor = necrosis area/whole area × 100% (necrosis area = the largest diameter × the smallest diameter of the necrosis area; whole area = the largest diameter × the smallest diameter of the area).

Signals of blood flow of CDFI were classified into four grades based on the criteria of Adler [26]: 0, no blood flow signals detected within the tumor; I, minimal blood flow (one or two dot-like or thin- and short-like blood flow signals detected within the tumor); II, moderate blood flow (up to three dot-like blood flow signals or one longer blood flow signal detected within the tumor); and III, abundant blood flow (more than five dot-like blood flow signals or two longer blood flow signals detected within the tumor).

### Microvessel density (MVD)

Tumor tissues were fixed immediately in 10% buffered formalin phosphate and embedded in paraffin. Immunohistochemical staining was performed using EnVision method. Briefly, the dewaxed, rehydrated sections (5 μm) were treated with 0.3% hydrogen peroxide in methanol for 15 min to block endogenous peroxidase activity, and then washed three times with Tris–HCl buffered saline (TBS). The sections were repaired in citric acid antigen repair solution (pH 6.0) for 40 min followed by treatment with primary antibody at 37°C for one night (anti-CD31 diluted for 1:100). After washed by TBS, the sections were treated with EnVision^TM^ diluted for 1:100 at 37°C for 45 min and washed with TBS. The sections were then stained with 3,3'-diaminobenzidine (DAB) under light-controlled microscope and counterstained with hematoxylin. Finally, the sections were dehydrated, fixed, and analyzed by light microscopy.

Microvessel density (MVD) of the tumor tissues was assessed through CD31 immunohistochemical analysis using antibodies to the endothelial marker CD31 and determined according to the method of Weidner et al. [27]. Briefly, the immunostained sections were initially screened at low magnification (40×) to identify hot spots, which are the areas of highest neovascularization. Any yellow-brown stained endothelial cell or endothelial cell cluster clearly separate from the adjacent microvessels, tumor cells, and other connective tissue elements was considered a single, countable microvessel. Within the hot spot area, the stained microvessels were counted in a single high-power (200×) field, and the average vessel count in three hot spots was considered the value of MVD. All counts were performed by three investigators in a blinded manner. Microvessel counts were compared between the observers and discrepant results were reassessed. The consensus was used as the final score for analysis [28].

### Side effects, quality of life, and survival

Another 30 mice were allocated randomly to groups a, b, and c as mentioned above (10 mice per group), with the aim to observe the different survival rates among the three groups. Side effects during the experiment, such as loss of appetite, weight loss, mental state, reaction to stimulation, behavior change, and ruffling of fur were recorded, as well as the survival time of every mouse.

### Statistical analysis

All statistical analyses were performed using SPSS 11.5 software package. TV, necrosis rate of tumor, and MVD were analyzed through one-way ANOVA, followed by the Student's *t* test. Data of tumor inhibitive rate was analyzed via Chi-square test. Survival curves were constructed according to the Kaplan–Meier method and statistical significance was determined via log-rank test. CDFI grade was analyzed using Kruskal–Wallis test. All P values were two-sided and P < 0.05 was considered the significant level of difference.

## Results

### PCR amplification of sKDR gene

**Figure 1:**
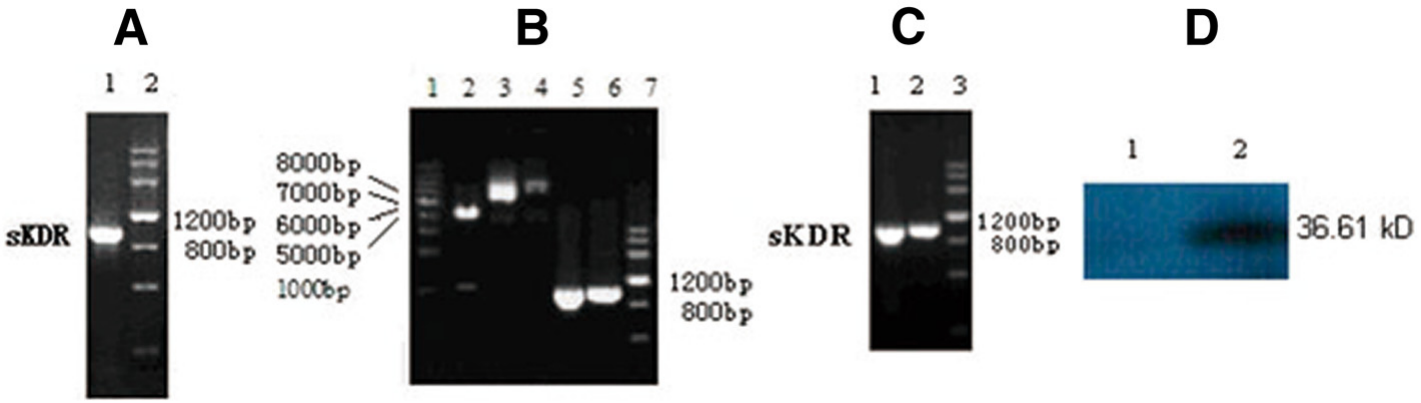
**Results of electrophoresis and Western blot.(A)** PCR products of sKDR. Lane 1: sKDR; Lane 2: DNA Marker III. **(B)** Endonuclease digestion and PCR products. Lane 1: DNA Ladder; Lane 2: digestion of recombinant plasmid pTRKH2-PsT/sKDR; Lane 3: recombinant plasmid pTRKH2-PsT/sKDR; Lane 4: plasmid pTRKH2-PsT; Lane 5: PCR products of sKDR gene from recombinant plasmid pTRKH2-PsT/sKDR; Lane 6: PCR products of sKDR gene from recombinant plasmid pcDNA3.1/sKDR; Lane 7: DNA Marker. **(C)** RT-PCR and PCR products from recombinant *Bifidobacterium infantis* of pTRKH2-PsT/sKDR. Lane 1: PCR products of sKDR gene from recombinant plasmid pTRKH2-PsT/sKDR; Lane 2: RT-PCR product of RNA purified from *Bifidobacterium infantis* with pTRKH2-PsT/sKDR; Lane 3: DNA Marker. **(D)** Western blot analysis of sKDR expression. Lane 1: pTRKH2-PsT/sKDR; Lane 2: pTRKH2-PsT.

The amplified products of sKDR gene were detected using 0.8% agarose gel electrophoresis. The results showed that a specific segment about 1 kb (Figure 1A) was observed, which was almost the size of the sKDR gene (981 bp).

### Identification of PCR-amplified products and enzyme-digested products and sequencing

The amplified products of pTRKH2-PsT/sKDR plasmid were detected using 0.8% agarose gel electrophoresis, where one fragment with size of approximately 1 kb was obtained (Figure 1B). The size was almost the same as that of the sKDR gene (981 bp).

The digested products of pTRKH2-PsT/sKDR plasmid were detected through agarose gel electrophoresis as well. The results showed that the recombinant positive pTRKH2-PsT/sKDR plasmid was about 8 kb. Two segments of approximately 981 bp and 6.9 kb extracted from the 8 kb recombinant plasmid (Figure 1B) were equal to the size of sKDR gene and pTRKH2-PsT plasmid, respectively.

Sequencing results showed that the size and sequence of the nucleotide acid of the inserted gene segment were completely consistent with the size and sequence of the nucleotide acid of sKDR gene (GenBank: AF063658). The full length of the inserted gene fragment was 981 bp. The sequence of two ends of the inserted gene was also consistent with *Bam*H I site and *Sal* I site. The sequencing identified that the foreign gene (sKDR gene) was correctly inserted into pTRKH2-PsT plasmid and transferred into *Bifidobacterium infantis.* The findings showed that the targeting gene therapy system of *Bifidobacterium infantis* was successfully constructed.

### Detection of sKDR gene expression of recombinant positive bifidobacterium infantis

The RT-PCR electrophoresis results of the positively transformed *Bifidobacterium infantis* showed that there was a specific segment of about 1 kb (Figure 1C), proving that the sKDR gene could replicate in recombinant *Bifidobacterium infantis.*

The expression of sKDR protein from positively transformed *Bifidobacterium infantis* was assayed via Western blot analysis using a mouse monoclonal antibody for sKDR. The results showed that there was a Western blot band from the *Bifidobacterium infantis*-transformed recombinant pTRKH2-PsT/sKDR plasmid (Figure 1D), which is consistent with the size predicted by Expasy proteomics tools (36.61 kD). In addition, there was no similar band from *Bifidobacterium infantis*-transformed recombinant pTRKH2-PsT plasmid (Figure 1D). This observation proved that sKDR could be expressed at the protein level in *Bifidobacterium infantis*-transformed recombinant pTRKH2-PsT/sKDR plasmid.

### Effect of final solution of recombinant positive *Bifidobacterium infantis* on the growth of HUVECs induced by VEGF

**Figure 2:**
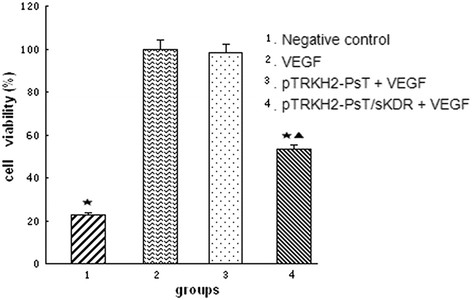
**Effect of*Bifidobacterium infantis*-mediated pTRKH2-PsT/sKDR delivery system on HUVECs.** 1: negative control; 2: VEGF; 3: VEGF and final solution of *Bifidobacterium infantis* transformed with pTRKH2-PsT plasmid; 4: VEGF and final solution of *Bifidobacterium infantis* transformed with recombinant pTRKH2-PsT/sKDR plasmid.^*^P < 0.05 vs. VEGF group; ^▲^P < 0.05 vs. negative control.

After treated with the final solution of recombinant positive *Bifidobacterium infantis* for 24 h, the growth of HUVECs induced by *VEGF* was significantly inhibited. Only a few grew along the wall, and many cells floated in the medium. The remaining cells underwent significant changes in morphology: the original shape was gone, the cytoplasm became rougher, the nucleus showed pycnosis, and the refraction decreased, demonstrating obvious cellular damages. By contrast, HUVECs treated with negative solution did not exhibit obvious morphologic changes compared with the control. Figure 2 shows that the cell viability of groups 2 and 3 had no significant difference (P > 0.05). However, the cell viability of groups 1 and 4 were significantly lower than that of group 2 (P < 0.05). The results showed that the VEGF could induce the growth of HUVECs significantly and that the final solution of recombinant positive *Bifidobacterium infantis* could inhibit the growth of HUVECs induced by VEGF.

### Specific targeting of recombinant *Bifidobacterium infantis* and sKDR expression in tumor tissue

**Figure 3:**
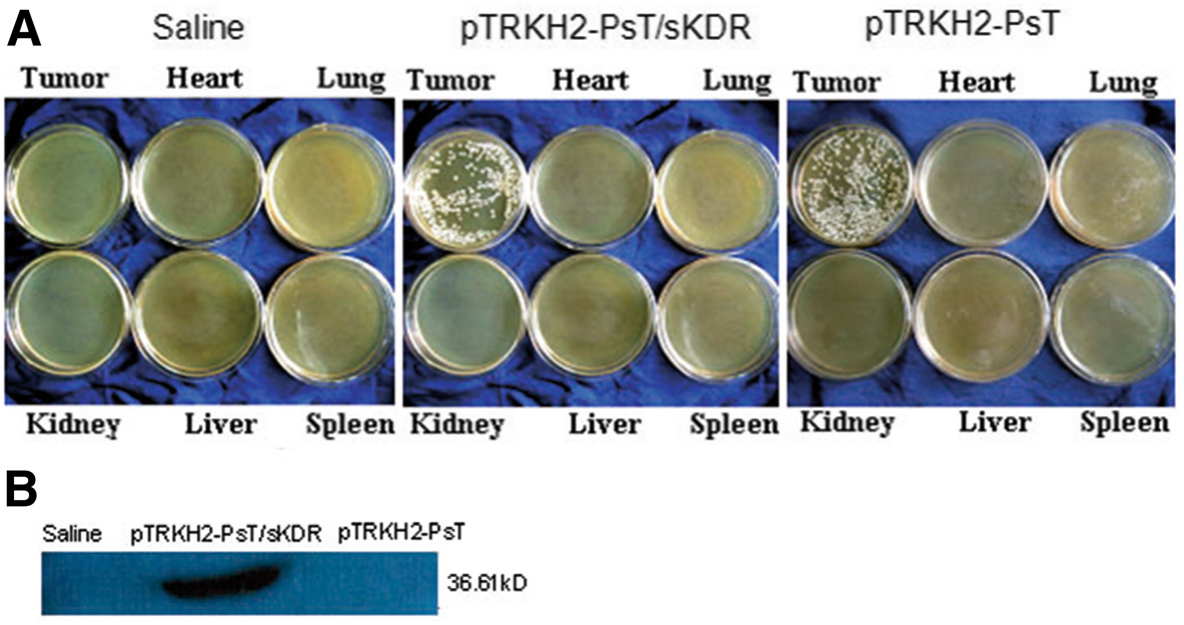
**Targeting property and sKDR expression.(A)** Targeting property of *Bifidobacterium infantis*; **(B)** Expression of sKDR in tumor tissue. Saline: saline control group; pTRKH2-PsT/sKDR: recombinant *Bifidobacterium infantis* containing pTRKH2-PsT/sKDR plasmid group; pTRKH2-PsT: recombinant *Bifidobacterium infantis* containing pTRKH2-PsT plasmid group.

Three days after incubation, a lot of white colonies were found in the medium culturing tumor tissue, whereas no colony was observed growing in the medium culturing other tissues, such as heart, lung, liver, spleen, and kidney (Figure 3A). These data showed that both the recombinant *Bifidobacterium infantis* containing pTRKH2-PsT/sKDR plasmid and pTRKH2-PsT plasmid had good targeting property to tumor tissues. Thus, *Bifidobacterium infantis* has the capability to specifically target the tumor tissue.

The findings in the Western blot (Figure 3B) showed that a band about 36.61 kD from *Bifidobacterium infantis* transformed the recombinant pTRKH2-PsT/sKDR plasmid. The same band was not shown in the saline control group or *Bifidobacterium infantis*-transformed recombinant pTRKH2-PsT.

### Tumor volume, tumor weight, and inhibitive rate of tumor

**Figure 4:**
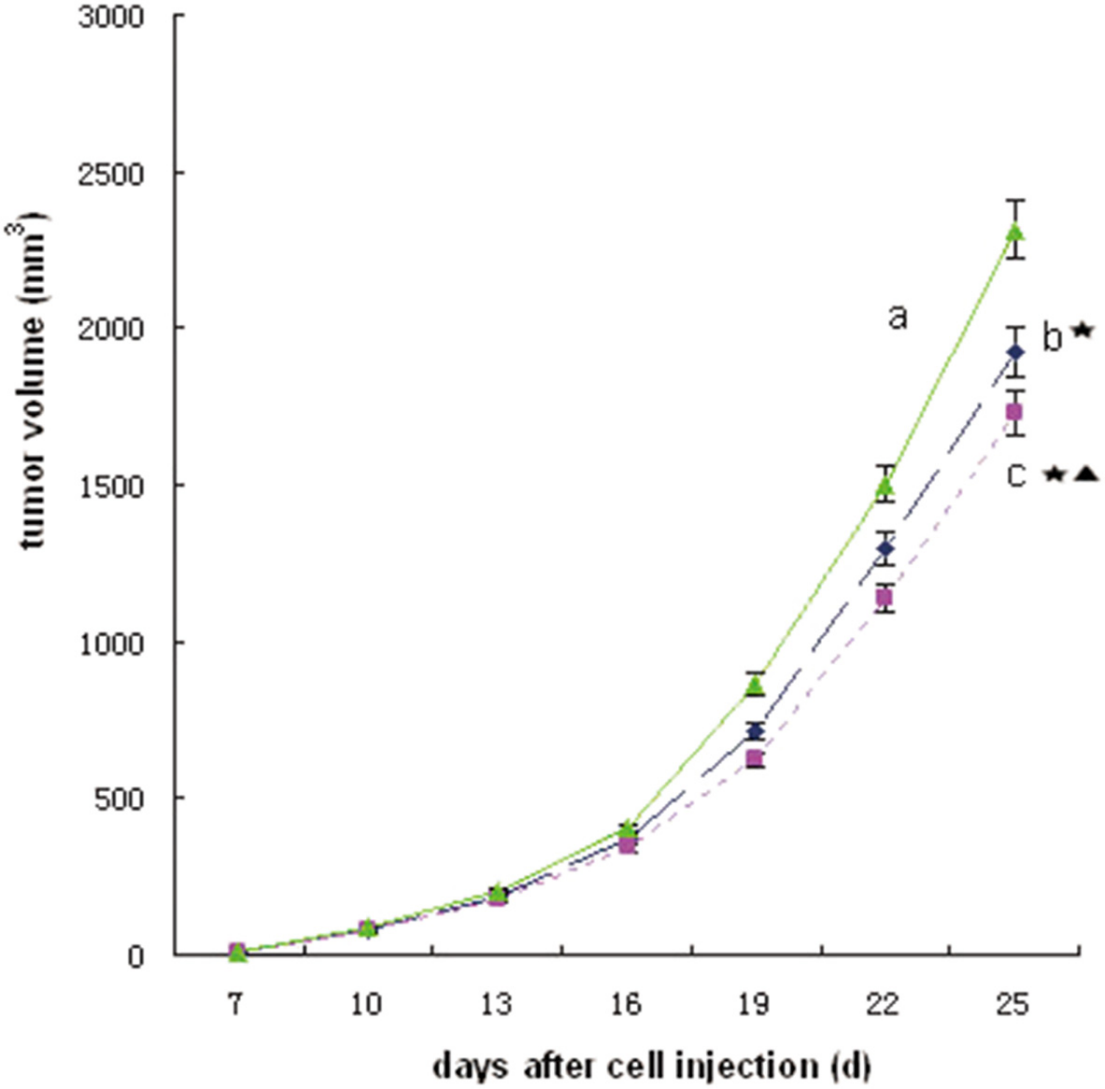
**Tumor growth curve.** a: saline control group; b: recombinant *Bifidobacterium infantis* containing pTRKH2-PsT plasmid group; c: recombinant *Bifidobacterium infantis* containing pTRKH2-PsT/sKDR plasmid group. ^*^P < 0.05 vs. group a; ^▲^P < 0.05 vs. group b.

**Figure 5:**
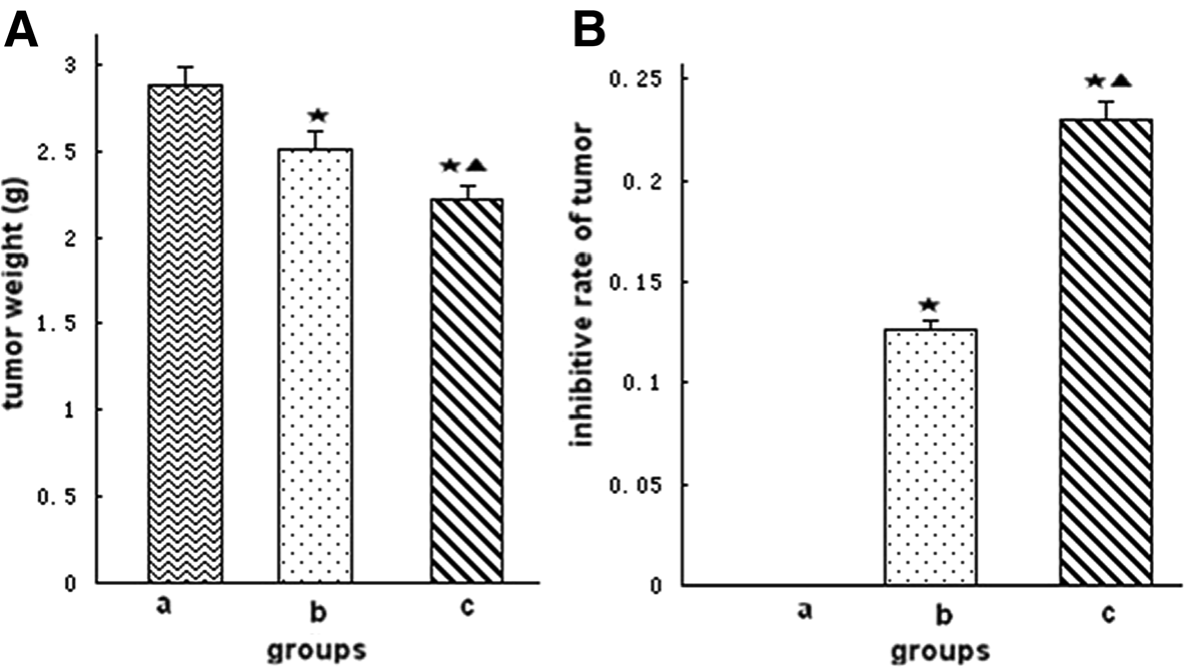
**Tumor weight and inhibitive rate of tumor.(A)** Tumor weight; **(B)** Inhibitive rate of tumor. a: saline control group; b: recombinant *Bifidobacterium infantis* containing pTRKH2-PsT plasmid group; c: recombinant *Bifidobacterium infantis* containing pTRKH2-PsT/sKDR plasmid group. ^*^P < 0.05 vs. group a; ^▲^P < 0.05 vs. group b.

The treatment began on the seventh day after the mice were injected with tumor cells. The volume and weight of tumors were recorded, and the acceleration curve was depicted (Figure 4). Figure 4 shows that the tumor volume of groups b and c were smaller than that of group a (P < 0.05), whereas the tumor volume in group c was lower than that in group b (P < 0.05). The data of tumor weight showed that the tumor weight of groups b and c were smaller than that of group a (P < 0.05), especially group c (P < 0.05) (Figure 5A). In addition, the inhibitive rate of tumor in groups b and c showed significant increase compared with that in group a (P < 0.05), and much more in group c (P < 0.05) (Figure 5B). All the results above showed that *Bifidobacterium infantis* containing pTRKH2-PsT/sKDR plasmid and pTRKH2-PsT plasmid could inhibit the growth of tumor. The *Bifidobacterium infantis* containing pTRKH2-PsT/sKDR plasmid exhibited more significant effect.

### Tumor necrosis rate

**Figure 6:**
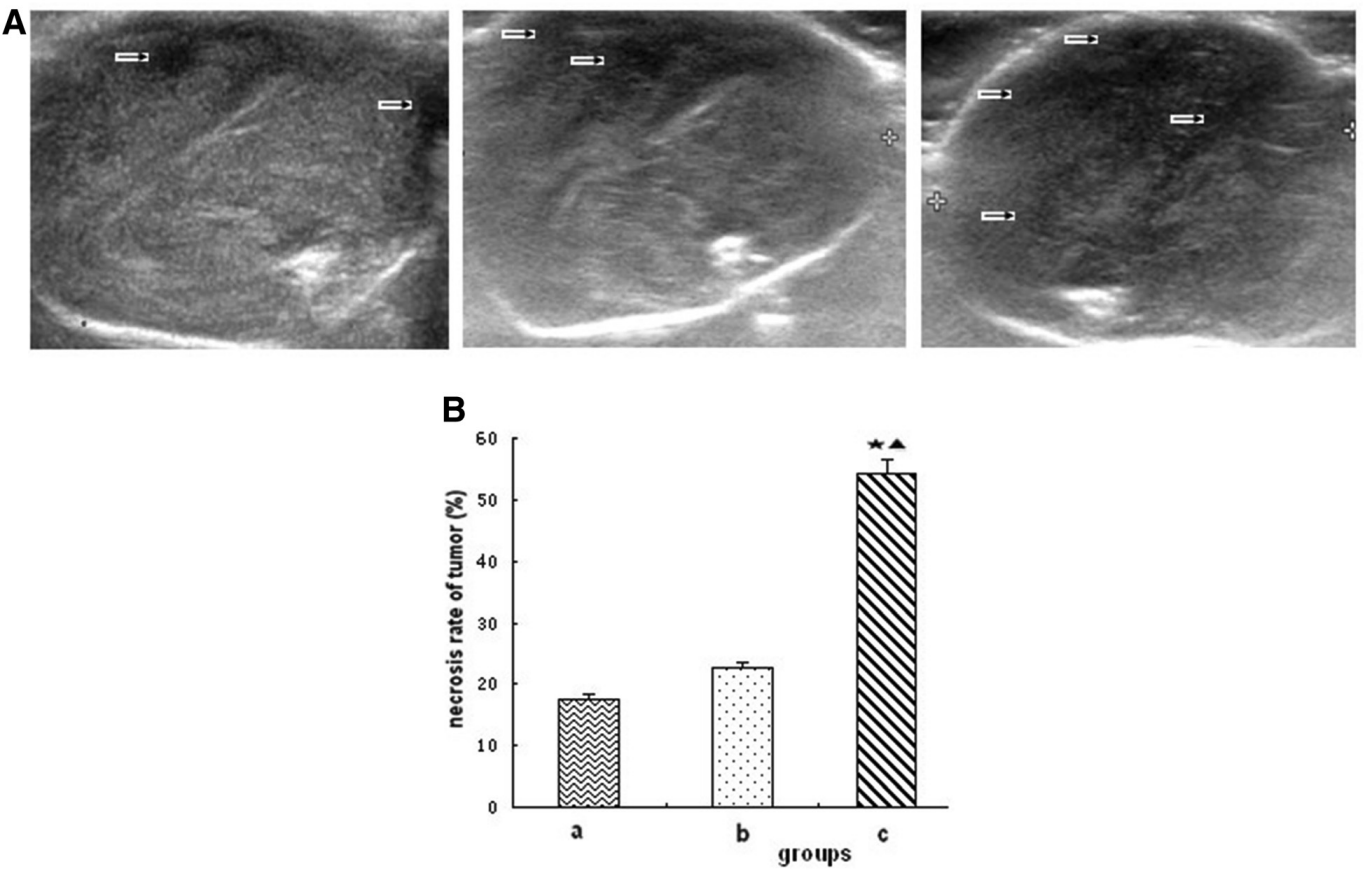
**Tumor necrosis.(A)** Ultrasound images of tumor; **(B)** Necrosis rate of tumor. a: saline control group; b: recombinant *Bifidobacterium infantis* containing pTRKH2-PsT plasmid group; c: recombinant *Bifidobacterium infantis* containing pTRKH2-PsT/sKDR plasmid group. The arrow shows the necrotic area. ^*^P < 0.05 vs. group a; ^▲^P < 0.05 vs. group b.

The necrotic areas of tumor showed low or no echo through the color Doppler ultrasound. Massive necrotic areas were observed in tumors of group c, with tumor necrosis rate of (54.25 ± 6.86) %. Small necrotic areas were observed in groups a and b, and the tumor necrosis rate were (17.62 ± 4.90) % and (22.75 ± 5.73) %, respectively. The tumor necrosis rate of group c was significantly larger than that of the groups a and b (P < 0.05) (Figure 6).

### Signal of blood flow in tumor

**Figure 7:**
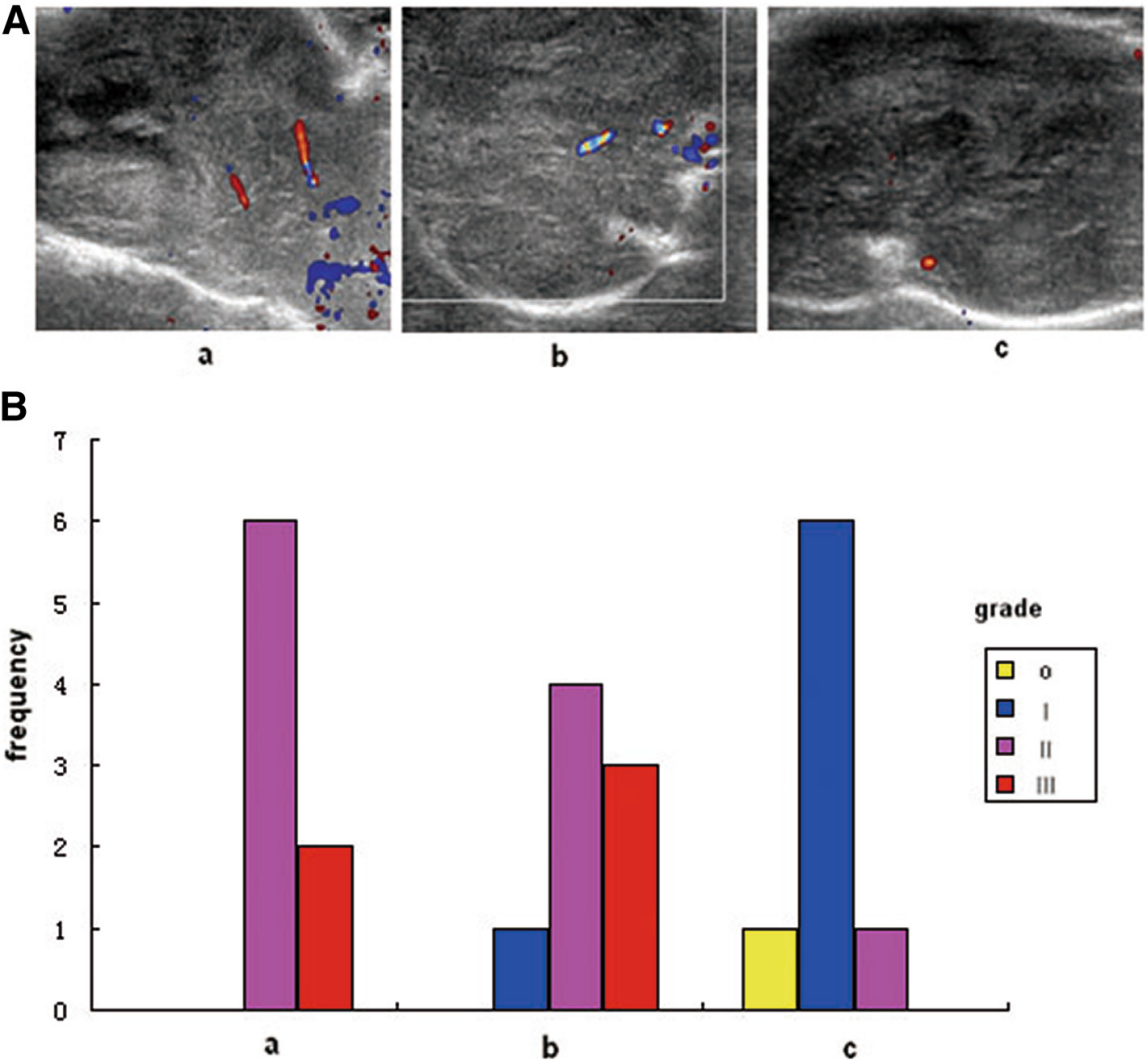
**Tumor blood flow.(A)** Blood flow image of tumor; **(B)** Classification of blood flow signals. a: saline control group; b: recombinant *Bifidobacterium infantis* containing pTRKH2-PsT plasmid group; c: recombinant *Bifidobacterium infantis* containing pTRKH2-PsT/sKDR plasmid group. Grade: 0, no blood flow signals were detected within the tumor; I, minimal blood flow (one or two dot-like or thin- and short-like blood flow signals detected within the tumor); II, moderate blood flow (up to three dot-like blood flow signals or one longer blood flow signals detected within the tumor); III, abundant blood flow (more than five dot-like blood flow signals or two longer blood flow signals detected within the tumor).

The color Doppler flow imaging showed that signals of blood flow in tumor were the worst in group c and the best in the control group (Figure 7). The signals of blood flow were mainly level 0–I (7/8) in the group c, whereas 100% at level II or level III (8/8) in the control group. The signals of blood in group b were intermediate between groups a and c.

### MVD

**Figure 8:**
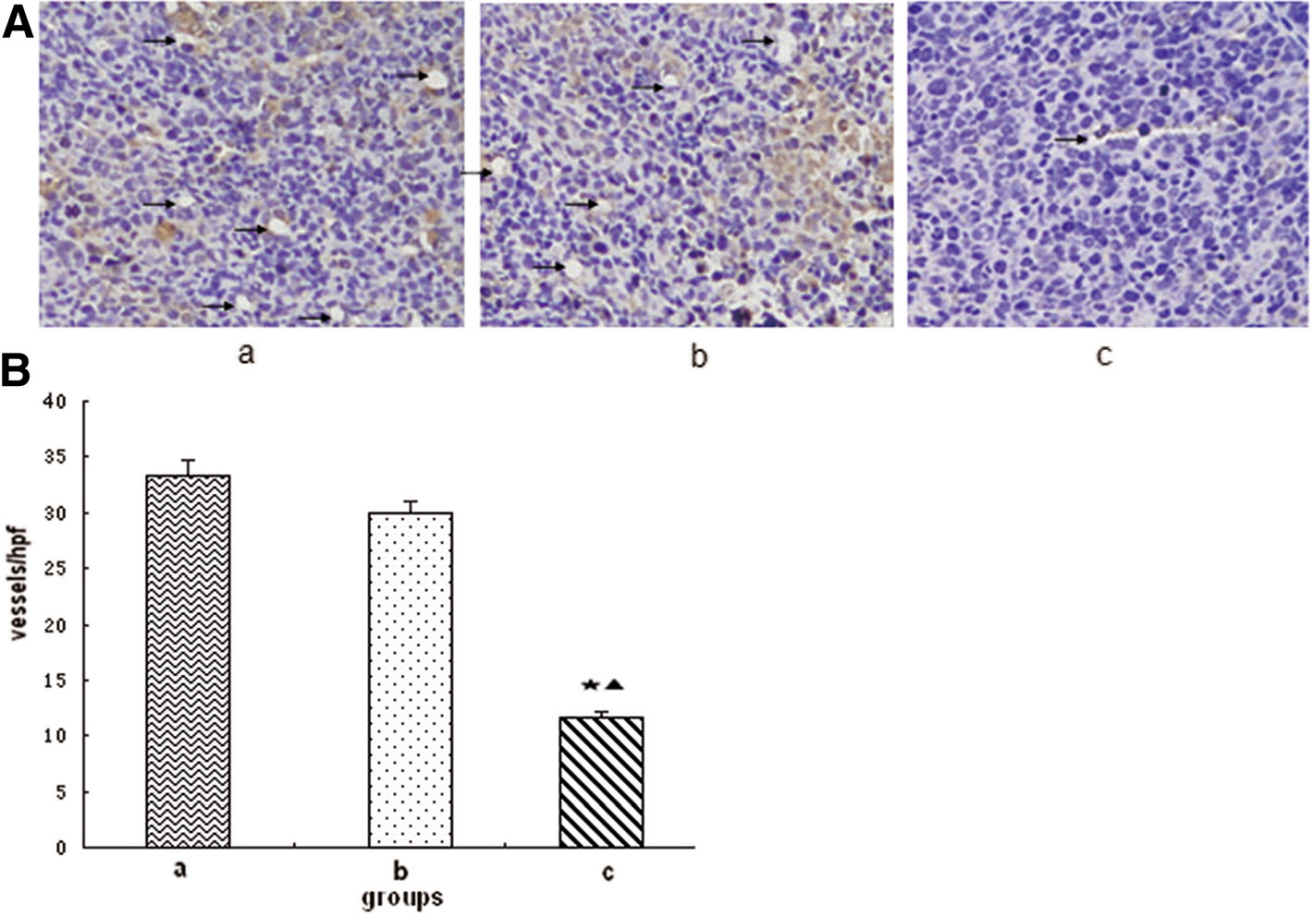
**Microvessel density (MVD).(A)** Photomicrograph of immunohistochemical staining of CD31 (200×); **(B)** MVD. a: saline control group; b: recombinant *Bifidobacterium infantis* containing pTRKH2-PsT plasmid group; c: recombinant *Bifidobacterium infantis* containing pTRKH2-PsT/sKDR plasmid group. ^*^P < 0.05 vs. group a; ^▲^P < 0.05 vs. group b. The arrows are directed to the vessels.

MVD was estimated by counting the number of the microvessels per high-power field (hpf) in the section with an antibody reactive to CD31 (Figure 8). The data showed that the MVD of group c was (11.6 ± 4.44)/vision (200×), whereas that of groups a and b were (33.4 ± 3.66)/vision (200×) and (30.0 ± 4.21)/vision (200×), respectively. The MVD in group c is significantly lower than that in groups a and b (P < 0.05), whereas no statistical significance was observed between groups a and b (P > 0.05).

### Side effects, survival quality, and analysis

Slight syndromes, such as bad appetite, stunted response, little activity, and colorless fur on the 12th day were observed on all the mice after the treatment, and became more evident over time. However, no significant differences were found among each group in mental status, appetite, weight, and so on.

**Figure 9:**
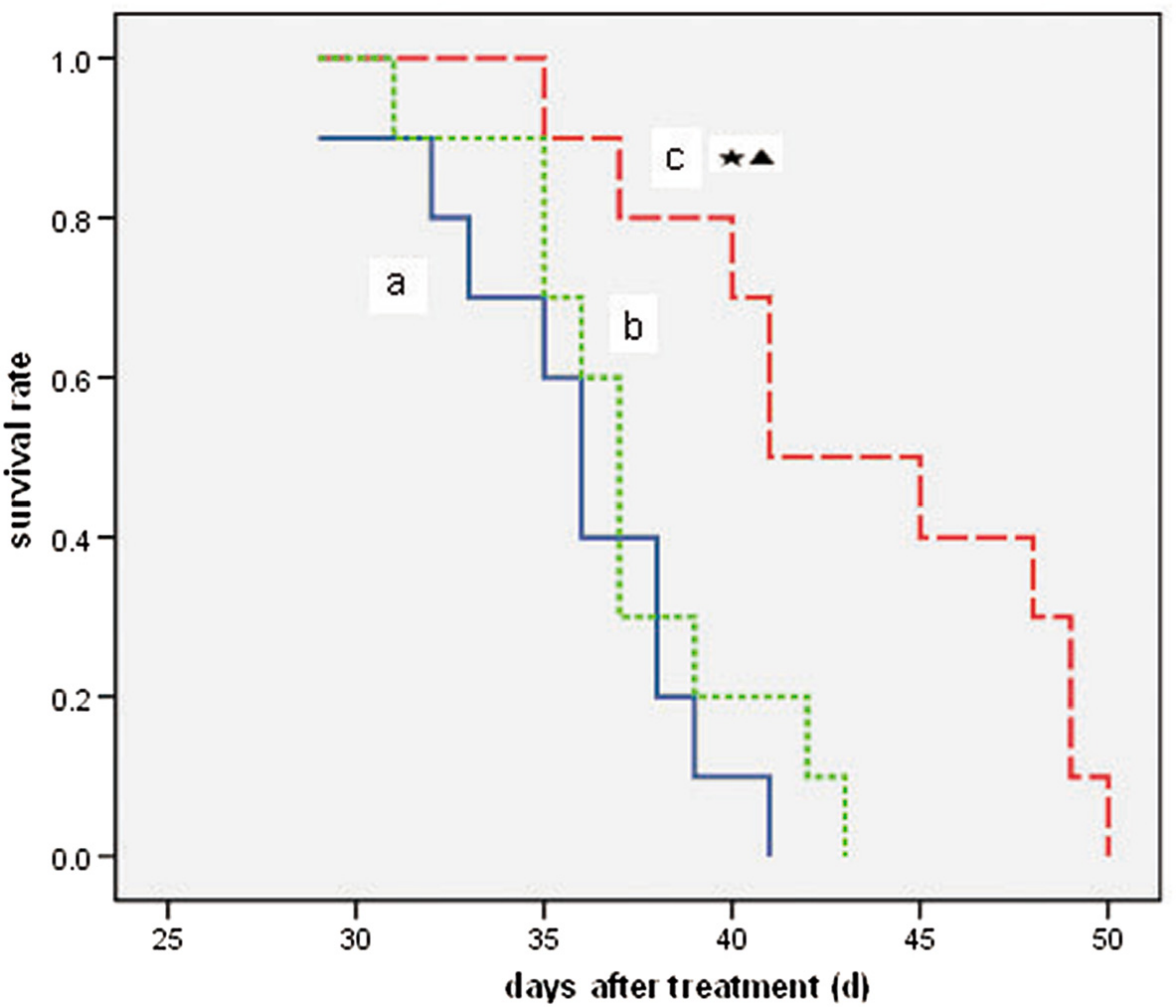
**Survival curve of tumor-bearing mice.** a: saline control group; b: recombinant *Bifidobacterium infantis* containing pTRKH2-PsT plasmid group; c: recombinant *Bifidobacterium infantis* containing pTRKH2-PsT/sKDR plasmid group. ^*^P < 0.05 vs. group a; ^▲^P < 0.05 vs. group b.

The mouse in the saline control group began to die on the 29th day after the treatment, and all them died on the 41st day because of tumor deterioration, excluding improper experimental manipulation by mice anatomy. However, 20% and 50% of mice in group b and group c were still alive on the 41st day, respectively (Figure 9). The survival in group c was significantly prolonged (P < 0.05) compared with that in groups a and b, whereas no statistical significance was found between groups a and b (P > 0.05).

## Discussion

Target gene therapy is one of the promising treatments of cancer due to its high effectiveness and safety [17,29]. However, a crucial difficulty in performing this therapy is the lack of specific delivery system [29,30]. Hypoxic regions are characteristics of solid tumors in rodents and occur with high frequency in many types of human tumors. Tissue oxygen electrode measurements taken in cancer patients show a median range of oxygen partial pressure of 10 mmHg to 30 mmHg in tumors, with a significant proportion of readings below 2.5 mmHg, whereas those in normal tissues range from 24 mmHg to 66 mmHg [31,32]. The hypoxic environment advocates a basis for using anaerobe as specific delivery carrier.

*Bifidobacterium infantis* is a kind of Bifidobacteria that is strictly anaerobic [22]. Many studies have found that they could specifically target to the anaerobic environment of the tumor center. Kimura et al. [33] injected *Bifidobacterium bifidum* intravenously into tumor-bearing mice to study its specific targeting property. They sacrificed the mice 48 and 96 hours after the injection and cultured the tumor tissue and other tissues. Kimura et al. [33] found that there were massive *Bifidobacterium bifidum* growing in the tumor tissue and no bacteria growing in other tissues, such as liver, spleen, kidney, lung, blood, bone marrow, muscle, and so on. Yazawa et al. [34] used *Bifidobacterium longum* as delivery system for gene therapy on tumor-bearing mice. They sacrificed 6 to 8 mice on 1, 2, 3, 4, and 7 days after the treatment,and cultured the lung, liver, spleen, kidney, heart, and tumor tissues. They found that *Bifidobacterium longum* could only specifically target the tumor tissue. In this study, the tumor and other tissues were also cultured after the mice were sacrificed. Many white colonies were observed in the medium culturing tumor tissue, whereas no colony was found growing in the medium culturing other tissues, such as heart, liver, lung, kidney, and spleen. Thus, *Bificobacterium infantis* has a very good targeting property to the anaerobic environment of tumor tissues.

*Bifidobacterium infantis* is a Gram-positive, domestic, and non-pathogenic bacteria found in the lower small intestine and large intestine of humans and some other mammalian animals. These intestinal organisms are believed to have health-promoting properties for their host, including increase of the immune response [35], inhibition of tumor growth, inhibition of carcinogenesis, and protection of the host against viral infection [36]. In addition, *Bifidobacterium* can be killed easily by antibiotics or in oxygen environment *in vitro* or *in vivo*[37]. Therefore, it is very safe to use *Bifidobacterium infantis* as delivery carrier. In this study, there were no obvious side effects on tumor-bearing mice after the intravenous delivery of *Bifidobacterium infantis* during the whole experiment process, which further proves its safety.

Antiangiogenesis therapy is one of the most important strategies for treating cancer [5,13]. Tumor angiogenesis is a complex of coordinated interactions between numerous proteins involved in different signaling pathways. Each step provides an opportunity for therapeutic intervention. Angiogenesis mediated by VEGF constitutes a new target for anticancer therapy, which has been explored through different forms of intervention aimed at blocking tumor neovascularization. Strategies to inhibit tumor angiogenesis include inhibition of angiogenic factor production and their receptors, inhibition of the VEGF signaling pathway, inhibition of the binding between VEGF and its receptors, and inhibition of intracellular transduction of the VEGF signal [10,13,31]. The KDR is VEGFR-2, which is one of the most important receptors for binding with VEGF. The structure of VEGFR-2 contains an intracellular part, a transmembrane part, and an extracellular part which are all essential for signal transporting [14]. The sKDR is the soluble form of the extramembrane part of VEGFR-2, which has same high affinity for VEGF but does not conduct signal. Thus, sKDR can bind with VEGF and compete with normal VEGFR-2 and can function as dominant negative by forming inactive heterodimers with membrane-spanning VEGF receptors [12,38]. To date, many studies have proven that the specific binding of sKDR with VEGF could significantly inhibit the blood vessel formation in tumor tissue, thereby inhibiting the growth, proliferation and migration of tumor [15,23,31,38].

In this study, *in vitro* experiment showed that the *Bifidobacterium infantis*-mediated sKDR prokaryotic expression system was successfully constructed and could express sKDR at both gene and protein levels. The products of this system could significantly inhibit the growth of HUVECs induced by VEGF. In addition, the *in vivo* experiment showed that *Bifidobacterium infantis* could specifically target to tumor tissue and express sKDR after intravenously injected. The blood flow signals and MVD of the tumor in group c were both lower than those of the other two groups, showing that the blood vessel formation in group c was significantly inhibited. At the same time, the tumors of mice in group c showed more serious necrosis rate and less growth rate. The mice in group c also exhibited longer survival time. All these results showed that the *Bifidobacterium infantis*-mediated sKDR prokaryotic expression system possessed anti-angiogenetic effects that further caused tumor necrosis and anti-tumor effects. Although this system could significantly reduce the MVD, increase the tumor necrosis and show greater tumor inhibitive rate, the effects on tumor volume and tumor weight seems to be week (Figures 4, 5A). We think the combined treatment of *Bifidobacterium infantis*-mediated sKDR prokaryotic expression system and chemotherapy or radiotherapy may be more effective and need further study in our future experiment.

In this study, *Bifidobacterium infantis* itself showed anti-tumor effects in some way. The results showed that the tumor volume in pTRKH2-PsT group was smaller than that in the control group, and the MVD and signals of blood flow in the tumor were less in pTRKH2-PsT group compared with the control group. The mechanism remains uncertain. *Bifidobacterium infantis* may be able to stimulate inflammatory response, induce the accumulation of immune cells, and promote the secretion of antitumor factors by macrophage. The structure of *Bifidobacterium infantis* cell wall had antitumor effects [22]. The local proliferation of *Bifidobacterium infantis* competing with the nutrition of tumor cells may contribute to its antitumor effects as well. However, the expression of sKDR may play a more significant role, based on a comparison of group b and group c.

## Conclusion

In this study, the *Bifidobacterium infantis*-mediated sKDR prokaryotic expression system was successfully constructed, and could express sKDR at both gene and protein levels. The system could not only inhibit the growth of HUVECs induced by VEGF, but also blood vessel formation and tumor growth on Lewis lung cancer mice model. This system showed specific and safe tumor targeting property. Thus, the *Bifidobacterium infantis*-mediated sKDR prokaryotic expression system may be a promising treatment method for cancer.

## Abbreviations

sKDR, Soluble kinase insert domain receptor; HUVECs, Human umbilicus vessel endothelial cells; LLC, Lewis lung cancer; VEGF, Vascular endothelial growth factor; TV, Tumor volume; MVD, Microvessel density; CDFI, Color Doppler flow imaging.

## Misc

Zhao-Jun Li and Hong Zhu contributed equally

## Competing interests

The authors declare that they have no competing interests.

## Authors' contributions

ZJL, HZ, FZ, SHM, JPH, CY and YH designed and performed the experiments, and contributed to manuscript writing. LCD and TGL performed pathology experiments. BUM and SHM performed ultrasound experiments. SHM and HZ analyzed the data. All authors read and approved the final manuscript.

## Pre-publication history

The pre-publication history for this paper can be accessed here:

http://www.biomedcentral.com/1471-2407/12/155/prepub
